# Prevalence of active trachoma and associated factors among children aged 1 to 9 years in rural communities of Lemo district, southern Ethiopia: community based cross sectional study

**DOI:** 10.1186/s12879-019-4495-0

**Published:** 2019-10-24

**Authors:** Endale WoldeKidan, Deresse Daka, Deresse Legesse, Tariku Laelago, Bealu Betebo

**Affiliations:** 1 Public health emergency management, Hadiya zone health department, Hossana, Ethiopia; 2 Department of medical laboratory, Hawassa University, Hawassa, Ethiopia; 3 Department of epidemiology, Hawassa University, Hawassa, Ethiopia; 4 Department of nursing, Wachemo University, Durame campus, Durame, Ethiopia

**Keywords:** Active trachoma, Prevalence, Children, Factors

## Abstract

**Background:**

Trachoma, caused by *Chlamydia trachomatis* is the leading infectious cause of blindness. It is transmitted via personal contact with infected ocular and nasal secretions by hands, fomites and eye- seeking flies. Active trachoma is more common among children aged 1 to 9 years. The objective of this study was determining the prevalence of active trachoma and associated factors among children aged 1 to 9 years in rural community of Lemo district.

**Methods:**

Community-based cross-sectional study was conducted from March to April, 2018 in rural community of Lemo district. Multistage sampling technique was used to select 589 study participants. Data were collected by using structured pre-tested questionnaire, physical examination and observation. Binocular loupe was used to identify active trachoma cases. The data were entered by using EPi-data version 3.1 and analyzed by SPSS. Binary logistic regression was used to assess factors associated with active trachoma. Variables with *p*-value < 0.05 in the multivariable analysis were used to declare significance of association.

**Result:**

Eighty seven (15.2%) children were positive for active trachoma. Absence of solid waste disposal pit (AOR = 2.20, 95% CI (1.12-4.37), do not use latrine as reported by respondent (AOR = 7.53, 95% CI (2.86-19.84), do not use soap for face washing as reported by respondent (AOR =2.3, 95% CI (1.32–4.12), washing face frequency as reported by respondent (AOR = 1.86, 95% CI (1.06–3.26), and family size greater than five (AOR = 1.96, 95% CI (1.06–3.67) were significantly associated with active trachoma.

**Conclusion:**

Active trachoma among children aged 1 to 9 years is high. Do not use latrine, do not use soap for face washing, and face washing frequency in a day as reported by respondents and family size were associated with active trachoma. Access to adequate water and sanitation can be important components in working towards eliminating trachoma as a public health problem. Therefore, prompt measures must be taken by concerned bodies to increase access to adequate water and sanitation facilities.

## Background

Trachoma, caused by particular serovars of *Chlamydia trachomatis (C. trachomatis)*, is the leading infectious cause of blindness [1]. It is transmitted in ocular and nasal secretions that are passed from person to person on fingers, fomites (such as bedding and washcloths) and eye-seeking flies (particularly *Musca sorbens*) [2]. *C. trachomatis* infection is associated with an inflammatory conjunctivitis known as “active trachoma”. Repeated episodes of active trachoma can result in eyelid scarring, which in some individuals leads to trachomatous trichiasis, in which one or more eyelashes are diverted to touch the eye [2].

*C. trachomatis* infections of the eye are commonest in young children. Children are the main reservoirs of infection. Active trachoma cases are more common among children aged 1 to 9 years [3]. Children are frequently infected with *C. trachomatis* due to their tendency to have close contact with others [4].

The diagnosis of active trachoma is a clinical diagnosis based on the WHO (world health organization) simplified grading system. Trachomatous inflammation - follicular (TF) and Trachomatous inflammation- Intense (TI) are indications of active trachoma and are usually found in children, but may occasionally occur in older persons [3, 5]. Each sign is individually graded as being absent or present. One or more signs can, and often do, occur together [3, 5].

Globally, an estimated 36 million people were blind, 216·6 million people had moderate to severe visual impairment and 188·5 million had mild visual impairment. The estimated number of blind people increased by 17·6%, from 30·6 million in 1990 to 36·0 million in 2015. The number of people with moderate and severe visual impairment also increased, from 159·9 million in 1990 to 216·6 million in 2015 [6]. Trachoma is responsible for blindness or visual impairments of about 1.9 million people [6].

About 165.1 million people existed in areas in which the TF occurrence in children aged 1–9 years was greater than or equal to 5% at some time during 2017. These people were fit for operation of the A, F and E components of the SAFE (Surgery, Antibiotic, Facial cleanliness and Environmental improvement) strategy for trachoma elimination. From 165.1 million people, 89% (146.3 million) were in WHO’s African region. Ethiopia accounted 69.8 million cases of WHO’s African regions. By 17 April 2018,157.7 million people were living in districts in which the TF prevalence was greater than or equal to 5% [7].

Different study findings in Ethiopia showed that the prevalence of active trachoma among children aged 1-9 years is high. The prevalence of active trachoma among children aged 1-9 was 29% in Amhara regional state [8], 12% in Dangla town [9], 25.2% in Kersa district, and 12.1% in Gondar zuria district of Ethiopia [10]. A region-wide study conducted in Southern nations, nationalities and peoples’ region showed the prevalence of active trachoma among children 1-9 years old 25.9%. The highest prevalence, 48.5% were observed in Amaro and Burji districts of Segen zone followed by Hadiya zone, which was 45% [11]. Study done in Zala district of Ethiopia showed that 36.5% of children aged 1 to 9 years had active trachoma [12]. Furthermore, the study carried out in Loma district of Ethiopia indicated that 75.4% of children had active trachoma [13].

The presence of active trachoma is associated with different factors. Income, educational status of parents, family size of households (HH), and age of children were factors associated wth active trachoma [4, 10, 14–17]. Washing face [8, 16], unclean face of child [12, 13], use soap for face washing [9, 16, 18], lack of access to pipe water [19], walking distance to fetch water [20], and lack of latrine [21, 22] were also associated with active trachoma. Moreover, disposing solid wastes and liquid on open field, using animal dung for cooking, and absence of separate room for animals were associated with presence of active trachoma [13, 18, 20, 23, 24].

The high burden of trachoma in Ethiopia in general and in Hadiya zone in particular calls for collecting a further district-specific data and comprehensive efforts for designing and expanding intervention programs. Prevention of trachoma related blindness requires a number of interventions. The WHO and their partners endorse SAFE strategy for trachoma control [4, 25]. Despite the implementation of SAFE strategy in study area, active trachoma still remains to be major public health problem. Moreover, in study area no report has been documented so far on prevalence and associated factors of active trachoma among children aged of 1-9 years. Therefore, this study determined the prevalence of active trachoma and associated factors among children aged 1 to 9 years in the rural community of Lemo district.

## Methods

### Study setting

The study was conducted in Lemo district, Hadiya Zone, Southern Ethiopia. The district is 230 km from Addis Ababa in the southwest direction. The district has two agro-climatic divisions: 10% kola (low land) and 90% woynedega (mid land). The projected total population of the district is 153,469 in 2018. Of this total population, children aged 1-9 years are 40,117. There are 33 rural and 2 urban Keble in the district. Concerning the health infrastructure, there are 7 government health centers, 33 health posts and 17 private health facilities.

### Study design and period

A community based cross-sectional study was carried out from March to April, 2018.

### Study participants

The source population of the study was children age 1 to 9 years. The study participants were children aged 1 to 9 years in selected HH. Children who had serious sickness and refused HH to participate in study were excluded from the study.

### Sample size

The sample size was calculated by using EPi–info software with a single population proportion with assumptions; 95% confidence interval, 0.05 margin of error, and prevalence of active trachoma, 36.7% [12], design effect 1.5 and 10% non-response rate. Based on the above assumption, the last sample size became 589 HH.

### Sampling procedure

The study participants were selected by using a multistage sampling technique. In the first stage, 10 Keble were selected from the total 33 rural Keble by simple random sampling method. The reason for selecting only 10 Keble was cost and feasibility issue. The final sample size was proportionally allocated to the selected ten Keble based on their number of HH with children 1-9 years. Systematic sampling technique was used to select HH from each selected Keble. To determine the interval of HH in selected Keble, k^th^value was used. In case when HH had more than one eligible child, one child was selected by lottery methods.

### Data collection processes and measurements

Data were collected by face-to-face interview, observation, and clinical eye examination by using pretested interviewer-administered structured questionnaire. The questionnaire was developed by reviewing different literatures. The questionnaire was first prepared in English and translated to Hadiyisa, and then translated back to English in order to ensure consistency (Additional file 1). Three integrated eye care worker (IECW) who have been trained on management and diagnosis of eye were assigned to perform eye examination. The IECWs are tropical data certified trachoma graders. Each eye was examined separately by using binocular examination loupes lenses (× 2.5). The examination of the eye was done by careful inspection of eye lashes, cornea, limbus, eversion of the upper lid and inspection of the tarsal conjunctiva. The reporting of eye examination results was based on the WHO grading system [3].

The dependent variable of the study was presence of active trachoma. It was assessed by TF and TI [3, 5]. TF is defined as the presence of five or more follicles of greater than 0.5 mm in diameter in the central part of upper tarsal conjunctiva. TI is pronounced inflammatory thickening of the tarsal conjunctiva that obscures more than half of the normal deep tarsal vessels. Active trachoma was defined as the presence of TF or TI according to the WHO Guidelines.

The independent variables of the study comprised socio demographic and economic factors, HH factors, environmental and child factors.

Variables assessed by questioning a HH resident included: sex of HH head, education status of HH head, occupation of HH head, family size and number children aged 1 to 9 years, marital status, religion, and ethnicity. Sex of child, age of child, frequency of face washing and using soap for face washing were also assessed by questioning a HH resident. Furthermore, time taken to fetch water, amount of water for domestic consumption, ownership of cattle and utilization of latrine were assessed by questioning a HH resident.

Variables assessed through observation by the research team included: number of rooms, separate room for cattle and cooking, presence of window in the cooking room, availability of latrine, type of latrine and distance of latrine from living house. Solid waste system availability, liquid waste disposal, presence of hand washing facility and feces seen in the compound were also assessed through observation by the research team. Moreover, cleanliness of child face and fingers, ocular discharge and nasal discharge were also assessed through observation by the research team.

#### Clean face

Absence of an ocular or nasal discharge on the face of child at the time of the visit.

#### Clean finger

Absence of dirty materials on fingers and fingers’ nail.

### Data quality control

Before beginning data collection, 2 days training was given for 3 data collectors, 3 trachoma examiners and 2 supervisors. Before the actual data collection, the examination of eye with questionnaire was pre-tested on 5% of final sample size in an adjacent Keble, which was out of study area. During the course of the data collection, data collectors were intensively supervised at each site. The completeness and accuracy of data was checked at the end of each day.

### Statistical analysis

The data were cleaned to check for its consistency, completeness and the existence of missed values. Then, it was entered into EPi-data and exported to SPSS version 20 for analysis. Descriptive findings were calculated by using frequencies, percentages, and summary statistics. Binary logistic regression model was fitted to assess factors associated with active trachoma. Variables with *P*-value < 0.25 in bivariate analysis were included in multivariable analysis to control confounding factors. Odds ratio at 95% CI was computed to show strength of association between dependent and independent variables. Variables with *P*-value < 0.05 in the multivariable analysis were used to declare significance of association.

## Results

### Socio demographic and economic characteristics

A total of 574 children aged 1 to 9 years were included in this study with response rate of 97.4%.

Majority, 521 (90.8%) HH heads were males. More than half HH heads, 311 (54.2%) attended primary schools. The occupation of HH head showed, 431 (75.1%) were farmers. One hundred eight six (32.4%) HH had more than two children aged 1-9 years. The median children less or equal to 9 years were 2 with minimum of 1 and maximum of 4. The family size was greater than five, for 384 (66.9%) HH. The median size of family members was 6 persons, with minimum of 3 and maximum of 12. Concerning to religion, 357 (62.2%) respondents were Protestants. Regarding to ethnicity, 558 (97.2%) were Hadiya. About, 549 (95.6%) HH heads were married (Table 1).

**Table 1:** Socio demographic and economic characteristics of HH heads in rural Lemo district, Southern Ethiopia, 2018

Variables	Frequency (*N* = 574)	Percent
Sex of HH head		
Male	521	90.8
Female	53	9.2
Educational status of HH head		
No formal education	137	23.9
Primary education	311	54.2
Secondary education	114	19.9
College and above	12	2.1
Occupation of HH head		
Farmer	431	75.1
Craftsman	30	5.2
Housewife	55	9.6
Merchant	52	9.1
Others^a^	6	1
Family size		
≤ 5 family members	190	3.1
> 5 family members	384	66.9
Number of 1 to 9 years children		
1-2 children	388	67.6
≥ 3 children	186	32.4
Marital status		
Married	549	95.6
Widowed	23	4
Divorced	2	0.3
Religion		
Orthodox	135	23.5
Protestant	357	62.2
Muslim	46	8
Catholic	36	6.3
Ethnicity		
Hadiya	558	97.2
Gurage	10	1.7
Kambeta	4	0.7
Silte	2	0.3

Other^a^ Daily labor.

### HH factors

In 360 (62.7%) HH, the family members live in one to two rooms. About, 223(38.9%) HH used 41-60 l water for domestic use. The median amount of water used for domestic use was 60 l with minimum of 20 and maximum of 100 l. Regarding to time taken on foot travel from water sources, 413 (72%) HH spent <=30 min to collect water. The median distance to travel to get water was 25 min with minimum of 10 and maximum of 60 min. Four hundred forty two (85.6%) HH kept domestic animals inside the living houses. Three hundred seventy four (65.2%) HH cooked their food inside the living rooms. About, 101(52.3%) cooking rooms had windows (Table 2).

**Table 2:** The HH condition in rural communities of Lemo District Ethiopia, 2018

Variables	Frequency	Percent
Rooms in HH, *N* = 574		
1-2 rooms	361	62.9
Three and above rooms	213	37.1
Time taken to fetch water from the source, *N* = 574		
≤ 30 min	413	72.0
> 30 min	161	28.0
Amount of water for domestic consumption, *N* = 574		
20-40 Liters	115	20.0
41-60 l	223	38.9
61-80 Liters	159	27.7
> 80 Liters	77	13.4
Cattle ownership, *N* = 574		
No	60	10.5
Yes	514	89.5
Separate room for cattle, *N* = 514		
No	440	76.7
Yes	74	12.9
Separate cooking room, *N* = 574		
No	374	65.2
Yes	200	34.8
Window for cooking room, *N* = 200		
No	99	48.7
Yes	101	52.3

### Environmental health related factors

Majority of HH, 561 (97.7%) had latrine. More than half, 318 (56.7%) latrine types was covered pit latrine. About, 548 (99.5%) family members utilized latrines. Only, 37 (6.6%) HH had larine with hand washing facility. Latrine distance from living house was greater than 10 m for 482(85.9%) HH. The median distance of latrine from living room was 15 m with minimum of 7 and maximum of 30 m. Merely, 168(29.3%) HH had solid waste disposal pit. Liquid waste disposal is near house for 411(71.6%) HH. In 14(2.4%) HH, feces were seen in the compound (Table 3).

**Table 3:** Environmental health related factors in Lemo district, 2018

Variables	Frequency	Percent
Availability of latrine *N* = 574		
No	13	2.3
Yes	561	97.7
Type of latrine *N* = 561		
Uncovered pit latrine	243	43.3
Covered pit latrine	318	56.7
Utilization of latrine, *N* = 561		
No	22	3.9
Yes	539	96.1
Latrine distance from living house, *N* = 561		
≤ 10 m	79	14.1
> 10 m	482	85.9
Hand washing facility, *N* = 561		
No	524	93.4
Yes	37	6.6
Solid waste disposal, *N* = 574		
No	406	70.7
Yes	168	29.3
Liquid waste disposal, *N* = 574		
Near house	411	71.6
Far from house	163	28.4
Feces in the compound, *N* = 574		
No	560	97.6
Yes	14	2.4

### Child factors

More than half, 296(51.6%) children were males. Regarding to age distribution of children, 372(64.8%) were aged 1-5 years. The mean age of children was 4.74 years. Three hundred fifty four (61.7%) children washed their face once a day. Children’s facial cleanness indicated that 457 (79.6%) children faces were unclean. Fifty one (8.9%) children had ocular discharge. Sixty (10.5%) children had nasal discharge. Regarding cleanness of children fingers, only 165 (28.7%) children fingers were clean. About, 330 (57.5%) children do not used soap for face washing (Table 4).

**Table 4:** Child features in rural communities of Lemo district Ethiopia, 2018

Variables	Frequency (*N* = 574)	Percent
Sex of child		
Male	296	51.6
Female	278	48.4
Age of child		
1-5 years	372	64.8
6-9 years	202	35.2
Frequency of face washing		
Once a day	361	62.9
Twice and more times a day	213	37.1
Face condition of child		
Unclean	125	21.8
Clean	449	78.2
Ocular discharge		
No	523	91.1
Yes	51	8.9
Nasal discharge		
No	514	89.5
Yes	60	10.5
Cleanness’ of the child finger		
No	409	71.3
Yes	165	28.7
Used soap for face working		
No	330	57.2
Yes	244	42.5

### Prevalence of active trachoma

The overall prevalence of active trachoma among children aged 1–9 years was 15.2%. Of total examined children aged 1–9 years, the prevalence of TF and TI was 13.6 and 1.6%, respectively.

### Factors associated with active trachoma

Variables with P-value less than 0.25 in bivariate analysis were transferred to multivariable logic regression analysis. The following variables were included into multivariable logistic regression model by using enter method: utilization of latrine, solid waste disposal pit, frequency of face washing, family size, use soap for face washing, rooms in HH, HH water consumption, time taken to fetch water age of child, separate cooking room, distance of latrine from living house and number of children age 1 to 9 years. However, in multivariable logistic regression analysis; utilization of latrine as reported by respondent, absence of solid waste disposal pit, face washing as reported by respondents, family size and do not use of soap for face washing as reported by respondents were found independently associated with active trachoma.

HH that did not have solid waste disposal pit were 2.2 times more likely to be affected by active trachoma than those HH that had solid waste disposal pit (AOR = 2.2, 95% CI (1.12-4.37).

Families that didn’t use latrine were 7.5 times more likely to be affected by active trachoma than those families who utilize latrine (AOR = 7.53, 95% CI (2.86-19.84).

Children who did not use soap for face washing were 2.3 times were more likely to develop active trachoma than children who used soap for face washing (AOR = 2.3, 95% CI (1.32 -4.12). Furthermore, children who washed their face once a day were 1.86 times more likely to have active trachoma than those who washed twice and more times (AOR = 1.86, 95% CI (1.06-3.26).

children living in HH with family size greater than five were about 2 times more likely to develop active trachoma than children living children living in HH less or equal to five family size (AOR = 1.97, 95% CI (1.06 -3.67) (Table 5).

**Table 5:** Factors associated with active trachoma among children aged 1–9 years, rural Lemo district, 2018

Variables	active trachoma		COR (95% C.I)	AOR (95% C.I)	*P*-value
	Yes (n (%)	No (n (%)			
Utilization of latrine					
No	12 (2.1)	10 (1.8)	7.78 (3.2-18.67)	7.53 (2.86-19.84)	0.000
Yes	72 (12.8)	467 (83.2)	1	1	
Solid waste disposal pit					
No	74 (12.9)	332 (57.8)	2.66 (1.4-4.9)	2.20 (1.12-4.37)	0.024
Yes	13 (2.3)	155 (27)	1	1	
Frequency of face washing					
Once a day	63 (11)	298 (51.9)	1.67 (0.363-0.995)	1.86 (1.06-3.26)	0.030
> = 2 a day	24 (4.2)	189 (32.9)	1	1	
Use soap for face washing					
No	65 (11.3)	265 (46.5)	2.48 (0.241- 0.68)	2.3 (1.32-4.12)	0.004
Yes	22 (3.8)	222 (38.7)	1	1	
Family size					
1-5	19 (3.3)	168 (29.3)	1	1	
Above 5	68 (11.8)	319 (55.6)	0.53 (1.1-2.3.24)	1.97 (1.06-3.67)	0.03

*COR* Crude odds ratio, *AOR* Adjusted odds ratio, *CI* Confidence interval

## Discussion

This study attempted to identify the prevalence and associated factors of active trachoma among children aged 1 to 9 years in rural communities of Lemo district. In current study the prevalence of active trachoma was 15.2%. The study also showed that the prevalence of TF was 13.6%. This greater than the criteria for elimination of trachoma as public health problem, which showed that the prevalence of TF < 5% in children aged 1-9 years old [26].

The prevalence of active trachoma in current study is different to the prevalence of studies done in different parts of Ethiopia [8, 9, 11, 12, 14, 17, 21]. The difference could be attributed to difference in study setting, the infrastructure and health care facilities. The other reasons for variations might be due to differences in utilization of sanitary services. The prevalence of active trachoma in current is lower than the study conducted in South nation’s nationalities people region, which showed 33.2% [27]. The lower prevalence of active trachoma in current study may be due to the implementation of SAFE strategy at population level.

The current study highlighted that HH that did not have solid waste disposal pit were more likely to be affected by active trachoma than those HH that had solid waste disposal pit. This result is in agreement with study conducted in Gondar zuria district of Ethiopia [23]. Study conducted in Daworo zone of Ethiopia also supported that solid wastes disposed on open field was associated with presence of active trachoma [13]. This could be explained by the fact that disposing a solid waste on open field attracts more vectors of trachoma.

Families that didn’t use latrine were more likely to be affected by active trachoma than those families who utilize latrine. This finding is consistent with a study conducted in Gonji Kolella district, and Gondar zuria district of Ethiopia [21, 23].

Children living in HH with family size greater than five were more likely to develop active trachoma than children living in HH less or equal to five family sizes. This finding is similar with studies conducted in Amhara region; Ethiopia [14, 15, 23]. The possible justification for this could be overcrowding living conditions among family members. This finding shows that limiting number of children is important things to prevent active trachoma.

The current study also identified that children who do not used soap for face washing were more likely to develop active trachoma than those who used soap for face washing. This is supported by previous studies conducted in Baso Liben, Laku town, Gondar zuria and Dangla, Ethiopia [9, 15–17]. Parents or caretaker should use or make child to use soap for face washing. Hygiene promotion is effective where there is good availability of water. It is even more effective when it promotes the use of soap or ash with hand and face washing [28]. Therefore, concerned bodies should do more on availing water and other sanitation facilities before promoting hygiene.

Furthermore, children who washed their face once a day had more chance to have active trachoma than children washed for two or more times in a day. This is consistent with previous studies done in Gondar zuria district [23], Gazegibella district [14] and Gonji Kolella district [21], and Wollo, Ethiopia [29]. The possible explanation for this could be the fact that frequent face washing can interrupts contact between children and trachoma vectors.

This study has some limitations which have to be taken into consideration while interpreting the findings. Methods used to measure distance from house to latrine, distance to a water source, and amount of water were not precise so that results could have been over or underestimated.

As being cross-sectional in the design, this study lacks to show temporal relationship between cause and effect.

Some variables such as cleanness of child face and fingers were taken based on observation during data collection, so that these variables could be subjected to observer’s bias. Face washing frequency of children could be subjected to social desirability bias.

The sample size used in current study was low for the purpose of estimating the prevalence of TF, as evidenced by WHO guidance, but the guidance was published after the study was done [30].

## Conclusion

The present study publicized high prevalence of active trachoma among children aged 1 to 9 years. Non-utilization of latrine, absence of solid waste pits, face washing habits of children, large family size and do not use of soap for face washing were factors associated with active trachoma. The district health office and other relevant stakeholders should cooperatively work on mass distribution of antibiotic to reduce the burden of active trachoma. Implementation of SAFE strategy should be strengthened by all concerned bodies.

Study done on sanitation and water supply coverage thresholds associated with active trachoma suggests getting sufficient water and sanitation can be vital components in working towards the 2020 target of eliminating trachoma as a community health problem. Therefore, concerned bodies should do more on availing enough water and sanitation facilities [31].

## Additional file

**Additional file 1:** English version of the questionnaires.

## Data Availability

The dataset used and/or analyzed during the current study are available from the corresponding author on reasonable request.

## Abbreviations

C: Chlamydia
HH: Household
IECW: Integrated Eye Care Workers
SAFE: Surgery, Antibiotic, Facial cleanliness and Environmental sanitation
SPSS: Statistical package for social science
TF: Trachomatous Follicular
TI: Trachomatous Inflammation
WHO: World health organization
