# Supplementary material for: Prevalence of active trachoma and associated factors among children aged 1 to 9 years in rural communities of Lemo district, southern Ethiopia: community based cross sectional study
Source: BMC Infect Dis. 2019 Oct 24;19:886. doi: 10.1186/s12879-019-4495-0 (PMC6813116; doi:10.1186/s12879-019-4495-0)
Supplement: Supplementary file 1 — Additional file 1: English version of the questionnaires. [file 12879_2019_4495_MOESM1_ESM.pdf]

English version of the questionnaires

Hawassa University College of Medicine and Public Health Science

Consent form

Department of Field Epidemiology

Good morning/Good afternoon. Thank you for your interest in talking with me today. My name is ______________ I am a member of team conducting a study to assess prevalence of active trachoma and associated factors among children aged 1 to 9 years. You are chosen to participate in this study. Your name will not be written on this form and will never be used in connection with any of the information you will tell me. You do not have to answer any question that you do not feel comfortable with, and you may end this task any time you want to. However, your honest answers to these questions will help us in better understanding of the situation of cases of active trachoma and its associated factors and will eventually help in designing and implementing appropriate intervention programs to alleviate related problem on active trachoma.

The interview will take 25-30 minutes.

Do you agree to participate in the study?   1. Yes   2. No

Participant ID No____________________

Name of interviewer_______________________ Signature _________Date ______________

Thank you very much

Name of kebele ________________________

Supervisor name and signature_______________________________________Date__________

S.#	Questions	Response	Remark
**1. Socio demographic condition of the head of the household**			
1.1	Sex of the head of the household	0. Male 1. Female	
1.2	What is your religion?	0. Orthodox<br>1. Protestant<br>2. Catholic<br>3. Muslim<br>4. Other : specify ---------	
1.3	What is your occupation?	0. Farmer<br>1. craftsman<br>2. Merchant<br>3.Government employee<br>4. Housewife<br>5. Other : specify---------	
1.4	What is your educational status?	0. No formal education<br>1. Primary education(1-8)<br>2. Secondary education(9-12)<br>3. Tertiary education and above	
1.5	What is your marital status?	0. Married 1. Single<br>2. Divorced 3 .Widowed	

1.6	What is your Ethnicity?	0. Hadiya 1. Kambata 2.silti 3. Gurage<br><br>4. other : specify---------	
1.7	Average monthly income of family in Ethiopian birr	--------	
1.8	Number of family members including you	------	
1.9	Number of children 1 to 9 years Old:	------	
**2. Household and environmental factors**			
2.1	Number of rooms in the living house:	-------	observation
2.2	How much water collected by family daily use in liters?	------- in liters	
2.3	For how long do you travel to get<br><br>Water for domestic use? in minutes	-------- in minutes	
2.4	Do you have latrine? If response No skip Q211	1. Yes 0. No	observation
2.5	If yes for above question, what type of latrine do you have?	0. Uncovered traditional pit latrine<br>1. Covered traditional pit latrine<br>2. VIP 3. other	observation
2.6	How much the distance of larine from living room?	------	observation
2.7	Do you use latrine? if the response is No skip to Q 2.11	1. Yes 0. No	
2.8	Do children wash their hands after use of	1. Yes 0. No	

	latrine?		
2.9	Do you have handing washing container after use of latrine?	1. Yes 0. No	observation
2.10	If yes for above question is there water in it?	1. Yes 0. No	
2.11	Do you have cattle? if the response is No, skip to Q 2.14	1. Yes 0. No	
2.12	Do you separate room for cattle?	1. Yes 0. No	observation
2.13	Do you have separate cooking room? if the response is NO, skip to Q 2.15	1. Yes 0. No	observation
2.14	Does the cooking room have window?	1. Yes 0. No	observation
2.15	Do you have solid waste disposal pit?	1. Yes 0. No	observation
2.16	Hoch much the disposal site from living room?	-------	
2.17	Where do you dispose liquid waste?	0. Nearby living house<br>1. Far away from the live house.<br>2. In the river or stream 3. Other : specify -------------	
2.18	Is a feces present in compound of house hold?	0. No 1. yes	observation
**3. Child's socio-demographic characteristics**			
3.1	Age of the selected child in years	-------	
3.2	Sex of the selected child	0 .Male 1. . Female	

**Child's health conditions**			
3.4	How often the selected child does wash his/her face?	0. Once daily<br>1. twice daily<br>2. Three or more times per day<br>3.once a week	
3.5	Does the child have ocular discharge?	1.yes 0.No	observation
3.6	Does the child have nasal discharge?	1.yes 0.No	observation
3.7	Child face condition	1. Clean face<br>0. Unclean face	observation
3.8	Absence of dirty materials on fingers and fingers' nail.	1.yes 0.No	observation
3.9	Does the selected child use soap when washing his/her face?	1.yes 0.No	
3.10	Result of the Eye examination for active trachoma	1.yes 0.No	
3.11	Yes, specify the stage of trachoma	-------	
